# Supplementary material for: Population genomics and haplotype analysis in spelt and bread wheat identifies a gene regulating glume color
Source: Commun Biol. 2021 Mar 19;4:375. doi: 10.1038/s42003-021-01908-6 (PMC7979816; doi:10.1038/s42003-021-01908-6)
Supplement: Supplementary file 2 — Supplementary Information [file 42003_2021_1908_MOESM2_ESM.pdf]

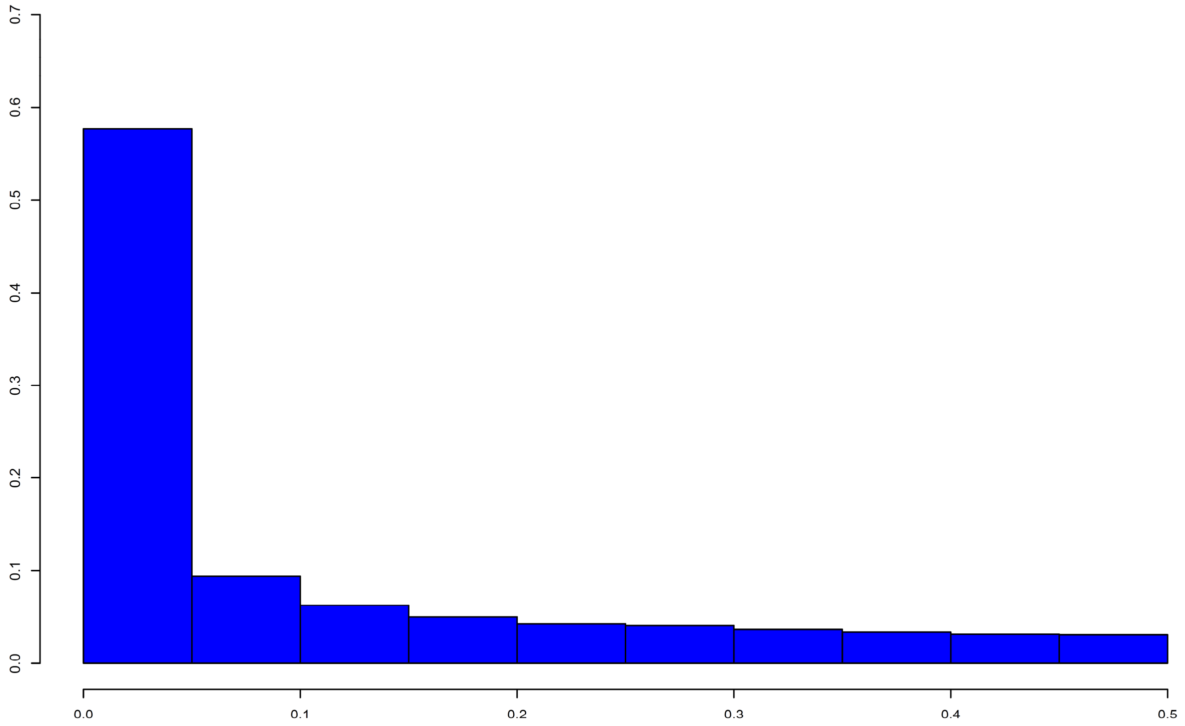

Supplementary Figure 1. Minor allele frequency (MAF) distribution of the genotyped spelt and bread wheat accessions. The minor allele frequency is shown on the X-axis and the density on the Y-axis.

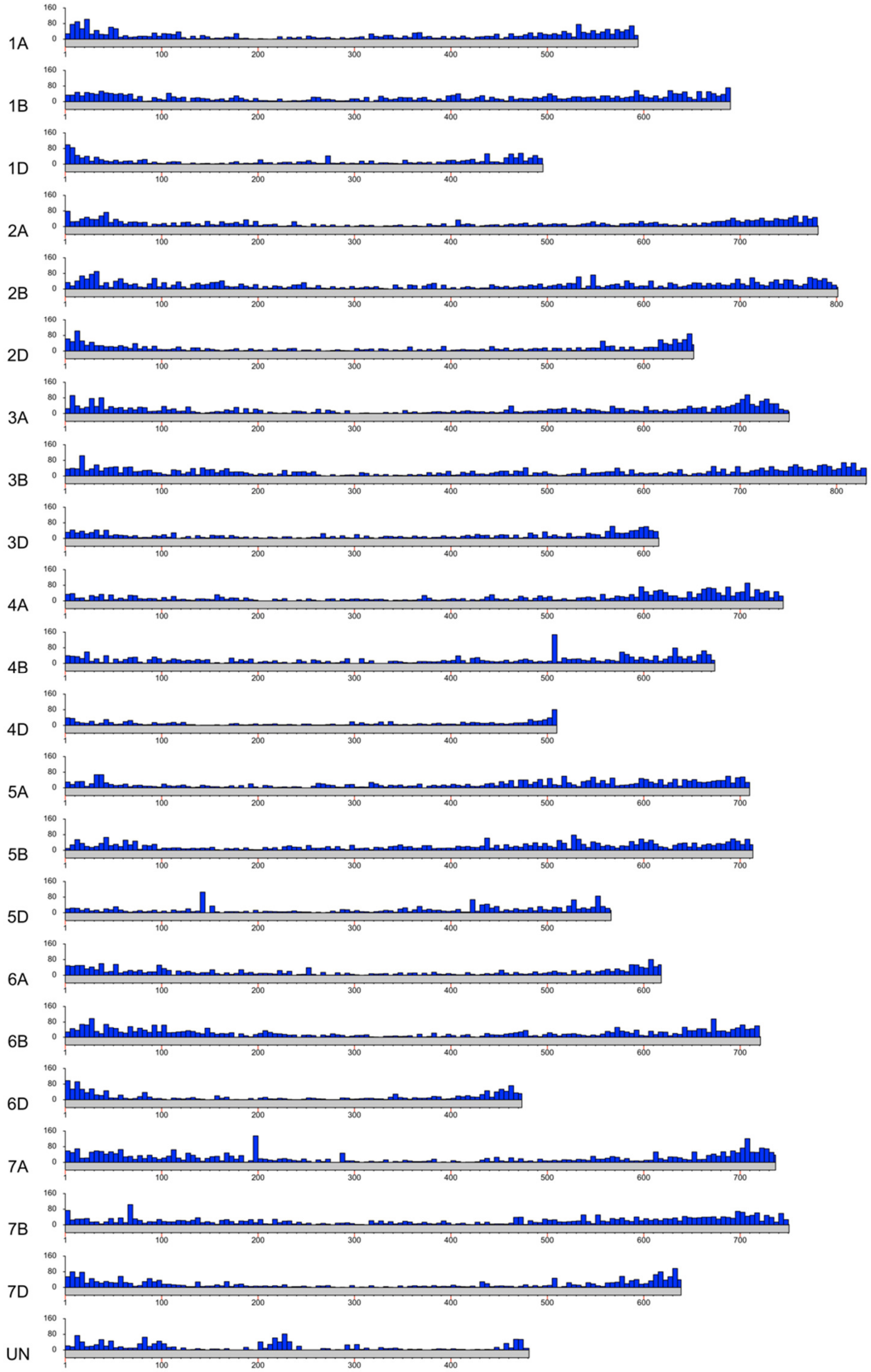

Supplementary Figure 2. Distribution of SNP markers across the different wheat chromosomes. Blue horizontal bars indicate the number of SNP markers in a sliding window of 5 Mb.

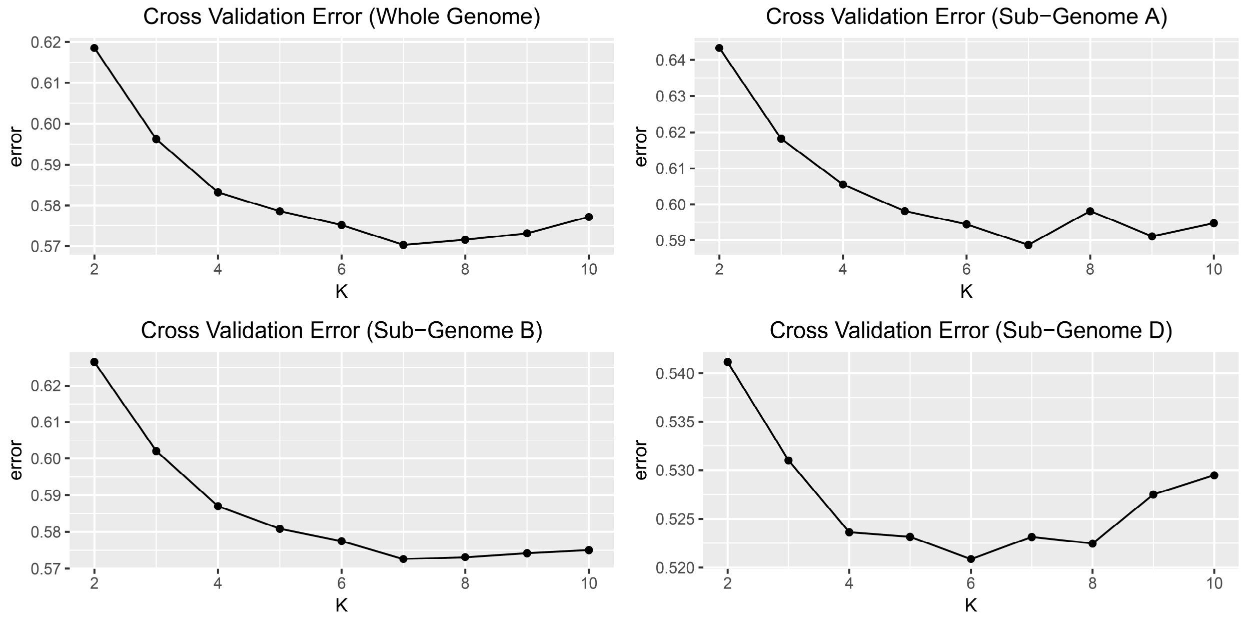

Supplementary Figure 3. Cross validation error plots for the ADMIXTURE analyses of the whole genome and subgenomes A, B and D, respectively.

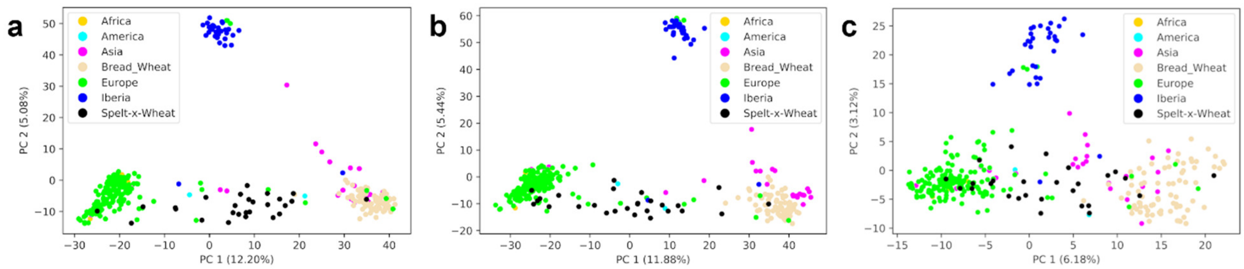

Supplementary Figure 4. Subgenome analysis of spelt and bread wheat accessions. Principal component analyses for the A subgenome (**a**), B subgenome (**b**) and D subgenome (**c**) are shown, respectively.

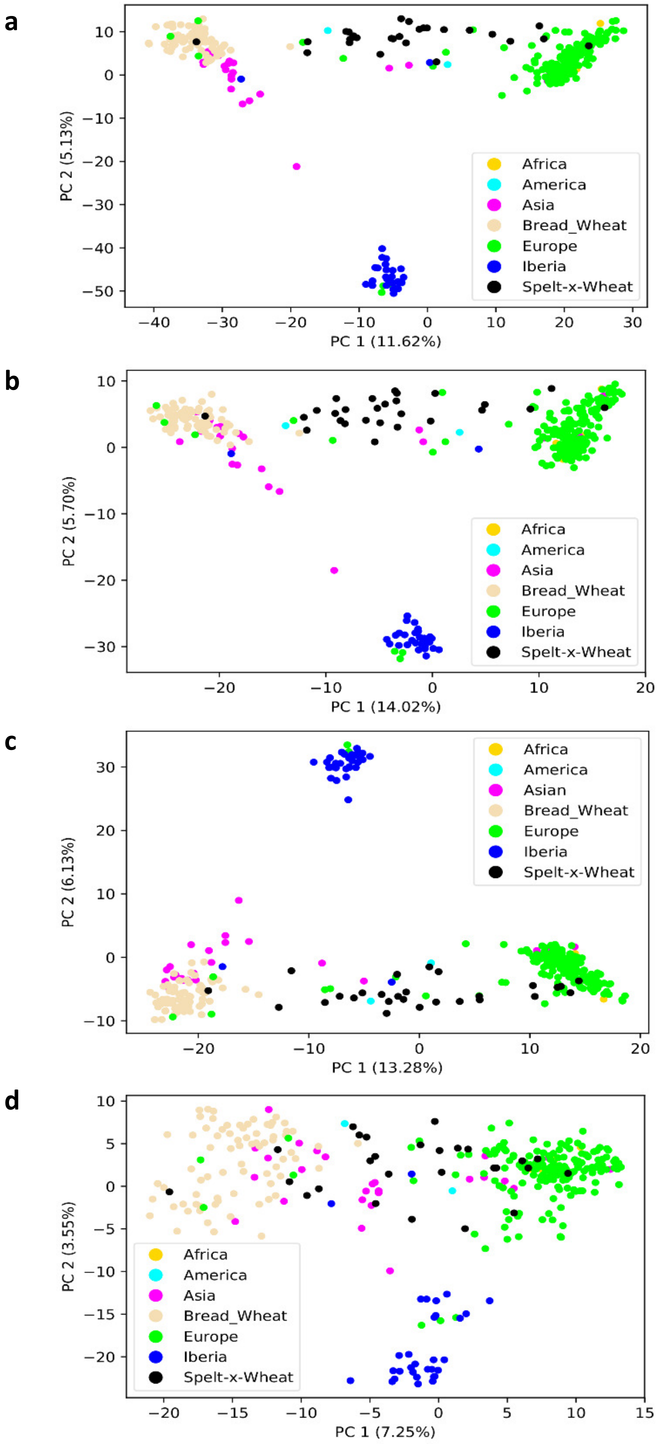

Supplementary Figure 5. Principal component analyses (PCA) with marker sub-sets. PCAs were constructed using 3,500 randomly extracted SNPs of the whole genome (**a**), A subgenome (**b**), B subgenome (**c**), and D subgenome (**d**), respectively.

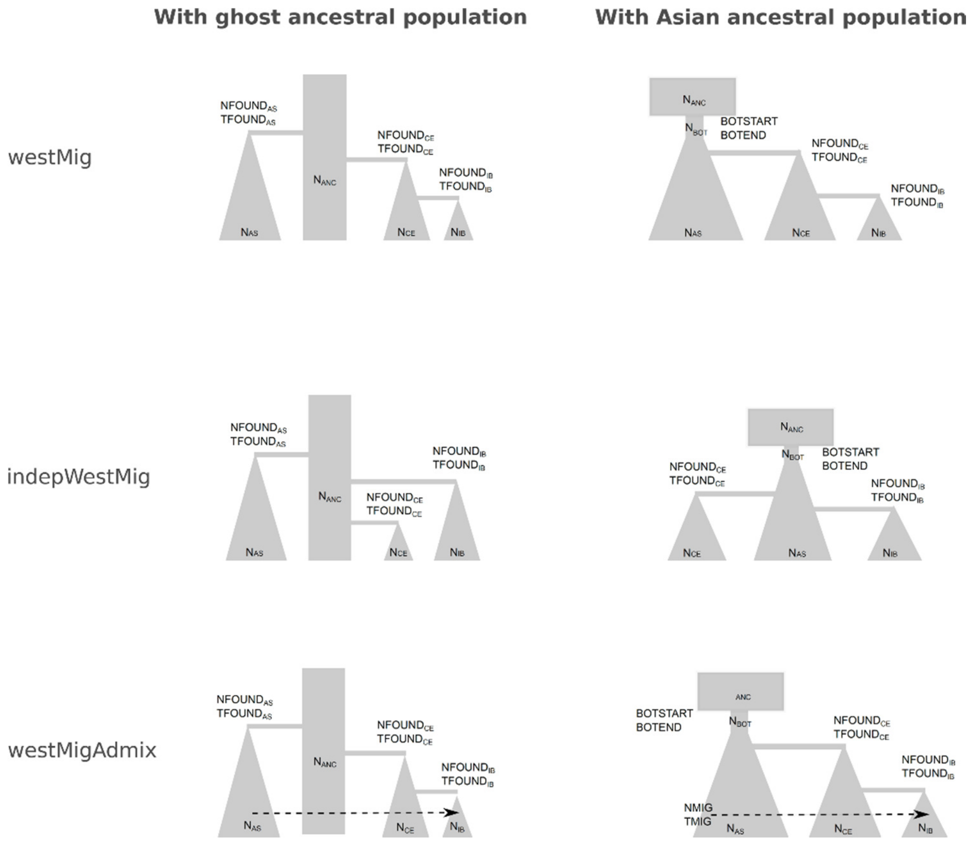

Supplementary Figure 6. The six demographic models that were compared in this study. Subscripts indicate population names (AS: Asia, CE: Central Europe, IB: Iberia, ANC: ancestral); N indicates population sizes, T indicates times. Detailed parameters are given in Supplementary Table 1. The terms ‘westMig’, ‘indepWestMig’ and ‘westMigAdmix’ refer to the evolutionary scenarios (i), (ii), and (iii) described in the main text, respectively.

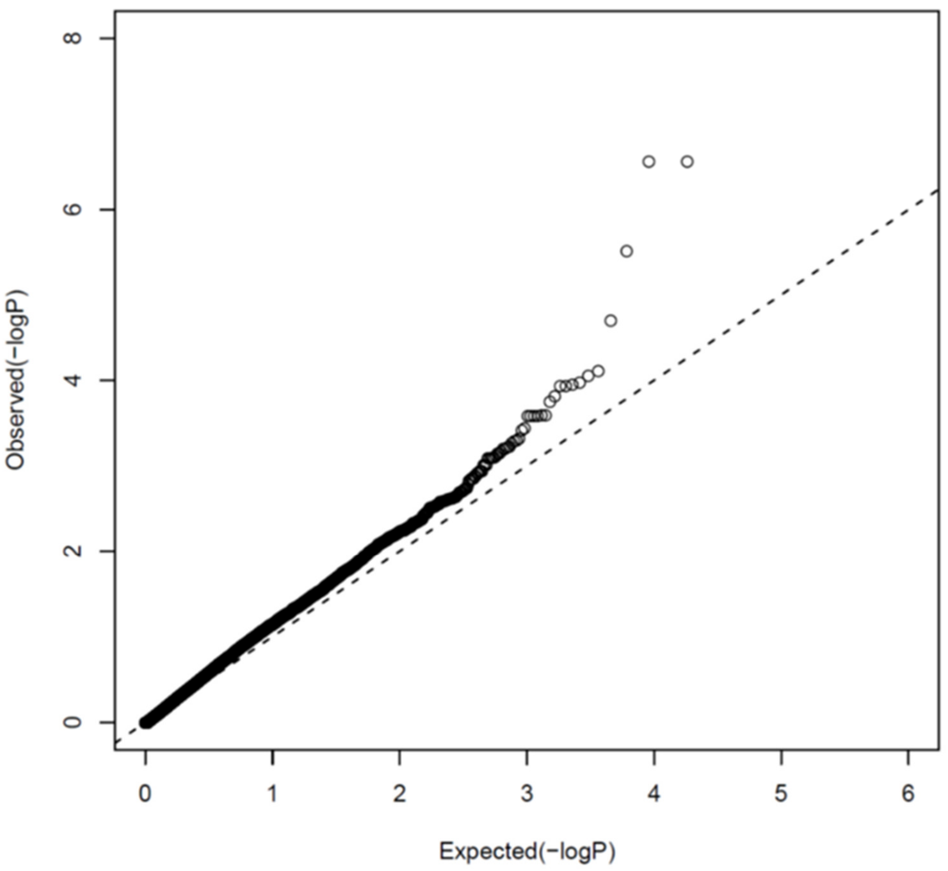

Supplementary Figure 7. Q-Q plot of observed versus expected –log10 p values of glume color GWAS using a logistic regression model.

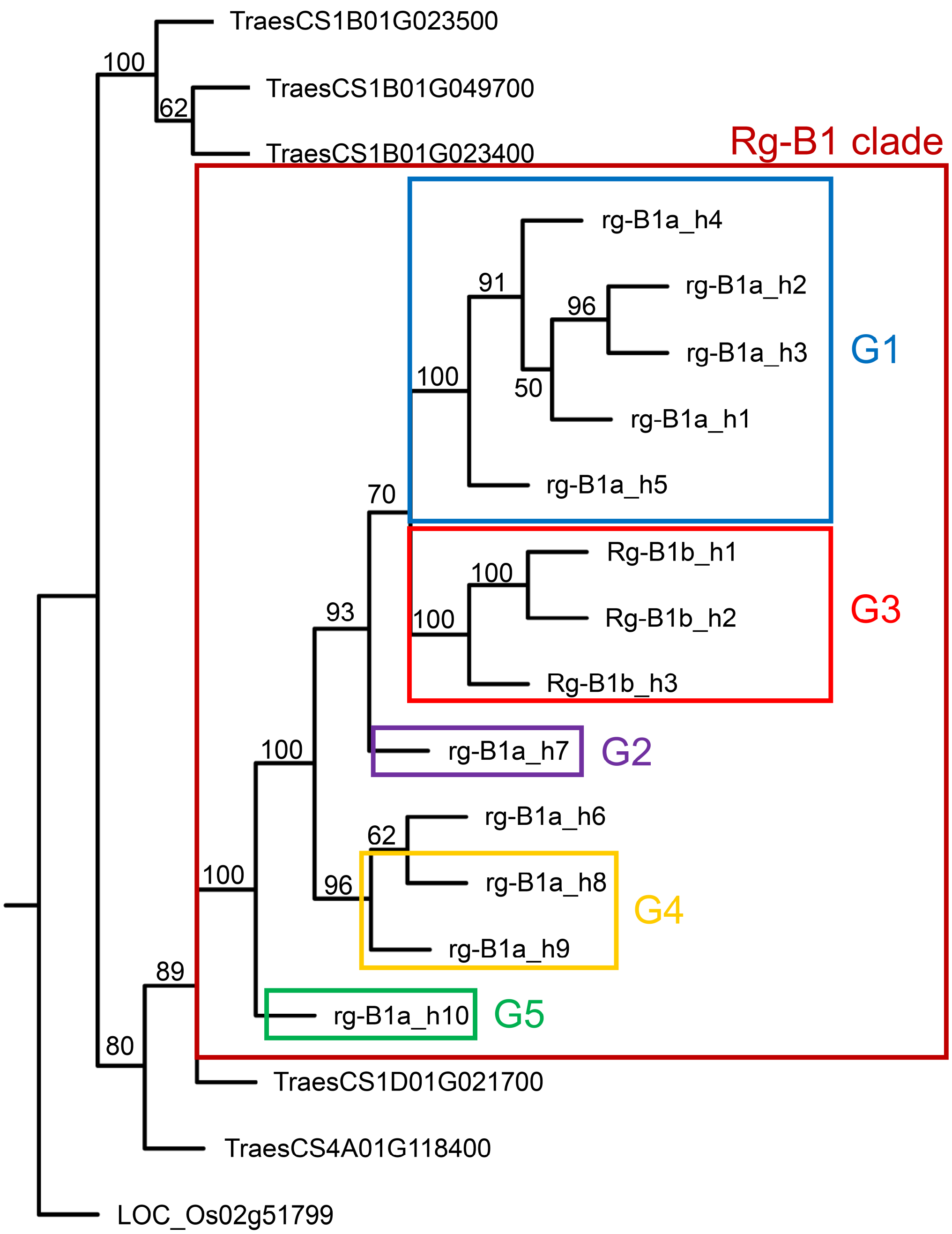

Supplementary Figure 8. Relationship between *Rg-B1 alleles*. Phylogenetic tree (neighbor joining) using the different *Rg-B1* cDNAs. The five closest homologs of TraesCS1B02G005200 (*rg-B1a_h1*) identified in Chinese Spring through BLAST have been included. LOC_Os02g51799 is a rice MYB transcription factor that has been used as outgroup. *rg-B1a_h6* is a chimeric allele with the 5' region belonging to group 1 and the 3' region to group 4. Based on the presence of the transposon in the second intron this allele was classified as group 1.

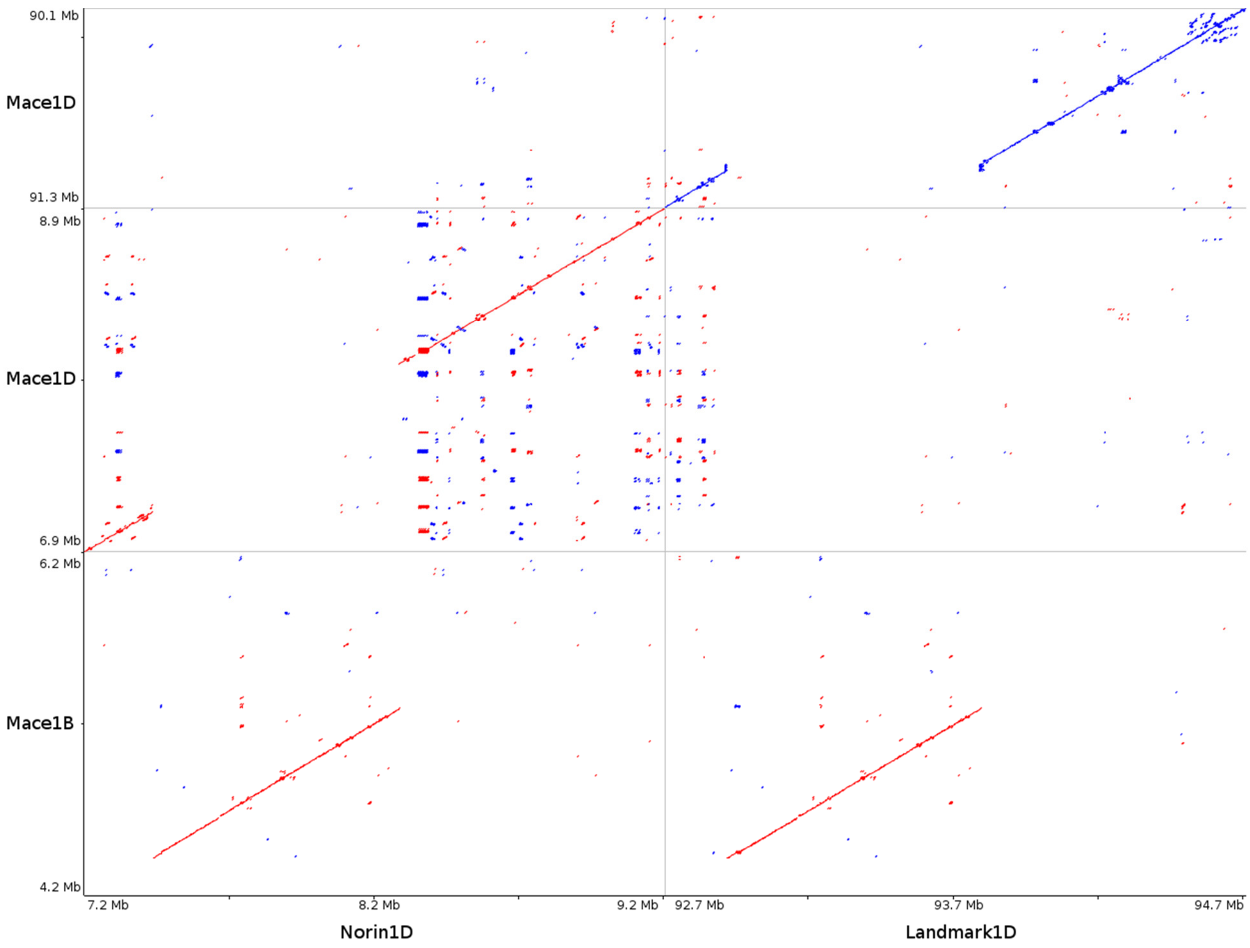

Supplementary Figure 9. Presence of *rg-B1a_h9* on chromosome 1D. Shown is a dot plot comparing segments of chromosomes 1B and 1D of Mace (vertical) to chromosomes 1D of Norin 61 and CDC Landmark. Norin 61 and CDC Landmark carry a~1 Mb segment on chromosome 1D that shows high similarity to a region of Mace chromosome 1B carrying the *rg-B1a_h9* allele.

```
               ----:----|----:----|----:----|----:----|----:----|----:----|
Rg-B1b_h1  MGRAPCCEKVGLKRGRWTAKEDDTLAKYIAGHGEGSWRSLPKNAGLLRCGKSCRLRWVNY   60
Rg-B1b_h2  MGRAPCCEKVGLKRGRWTAKEDDTLAKYIAGHGEGSWRSLPKNAGLLRCGKSCRLRWVNY   60
Rg-B1b_h3  MGRAPCCEKVGLKRGRWTAKEDDTLAKYIAGHGEGSWRSLPKNAGLLRCGKSCRLRWVNY   60

               ----:----|----:----|----:----|----:----|----:----|----:----|
Rg-B1b_h1  LRDGVRRGNFSKEEDDLIAELHATFGNRWSLIASHLPGRTDNEIKNYWNAHLSRQIHSFR  120
Rg-B1b_h2  LRDGVRRGNFSKEEDDLIAELHATFGNRWSLIASHLPGRTDNEIKNYWNAHLSRQIHSFR  120
Rg-B1b_h3  LRDGVRRGNFSKEEDDLIAELHATFGNRWSLIASHLPGRTDNEIKNYWNAHLSRQIHSFR  120

               ----:----|----:----|----:----|----:----|----:----|----:----|
Rg-B1b_h1  RMYTTGKETTITIDMNKLSAASKRRGGRAPRSSTKKQPVLEPTKSMECSRLVGDMSSSTS  180
Rg-B1b_h2  RMYTTGKETTITIDMNKLSAASKRRGGRAPRSSTKKQPVLEPTKSMECSRLVGDMSSSTS  180
Rg-B1b_h3  RMYTTGKETTITIDMNKLSAASKRRGGRAPRSSTKKQPVLEPTKSMECSRLVGDMSSSTS  180

               ----:----|----:----|----:----|----:----|----:----|----:----|
Rg-B1b_h1  SLPQGNSETMSDQNQPINIITNDTPCSEEPMVMDPIDLNGGFLEPNYVYNSMDQIGILQE  240
Rg-B1b_h2  SLPQGNSETMSDQNQPINIITNDTPCSEEPMVMDPIDLNGGFLEPNYVYNSMDQIGILQE  240
Rg-B1b_h3  SLPQGNSETMSDQNQPINIITNDTPCSEEPMVMDPIDLNGGFLEPNYANNSMDQIGILQE  240

               ----:----|----:----|----:----|----:----|----:----|----:----|
Rg-B1b_h1  SGEIGALLSSINDMPASVLTGIEHGGHSPRVEDLLDMDWEGFASHLCDQPAQNDILQTAK  300
Rg-B1b_h2  SGEIGALSSSINDMPASVLTGIEHGGHSPRVEDLLDMDWEGFASHLCDQPAQNDILQTAK  300
Rg-B1b_h3  SGEIGALLSSINDMPASVLTGIEHGGHSPRVEDLLDMDWEGFASHLCDQPAQNDILQTAK  300

               ----:----|----:----|----
Rg-B1b_h1  PQATTCSESDKLESFVSWLLSDAS                                     324
Rg-B1b_h2  PQATTCSESDKLESFVSWLLSDAS                                     324
Rg-B1b_h3  PHATTGSESDKLESFVSWLLSDAS                                     324
```

Supplementary Figure 10. Alignment of deduced amino acid sequence of *Rg-B1* alleles. Amino acid sequence alignment of Rg-B1b_h1, Rg-B1b_h2 and Rg-B1b_h3. Amino acid residue highlighted in brown (L248S) indicates the Rg-B1b_h2 variant. Amino acid residues highlighted in green (V228A, Y229N, Q302H and C306G) indicates the Rg-B1b_h3 variant.

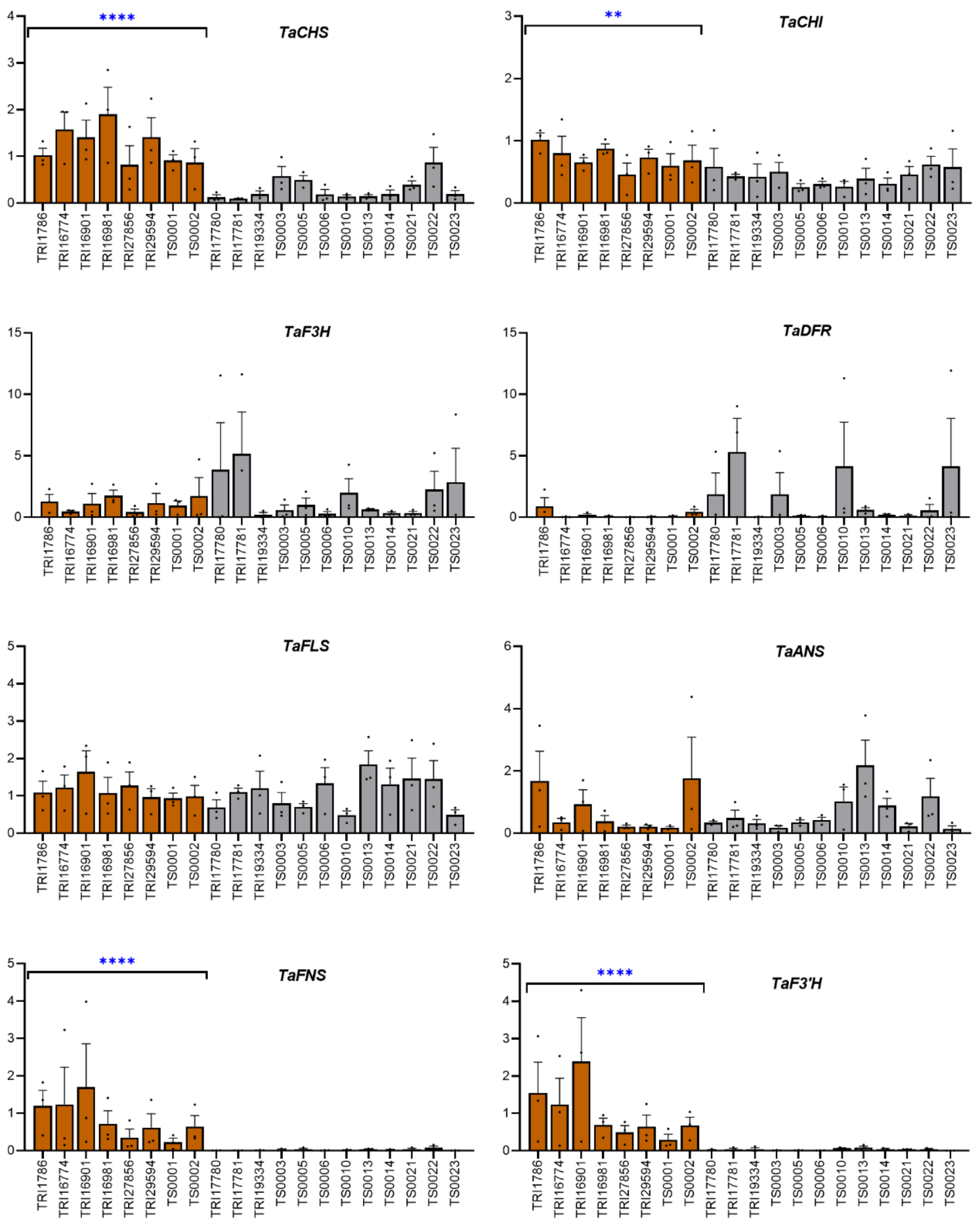

Supplementary Figure 11. Expression profile of structural genes of the flavonoid biosynthesis pathway in spelt glumes. Red bars represent red glume spelt accessions, grey bars represent white glume spelt accessions. Error bar represents the mean ± standard error of three biological replicates. Asterisks indicate significant differences between the red glume and white glume accessions (** = $p<0.01$; **** = $p<0.001$, two-tailed t-test, n = three biological replicates).

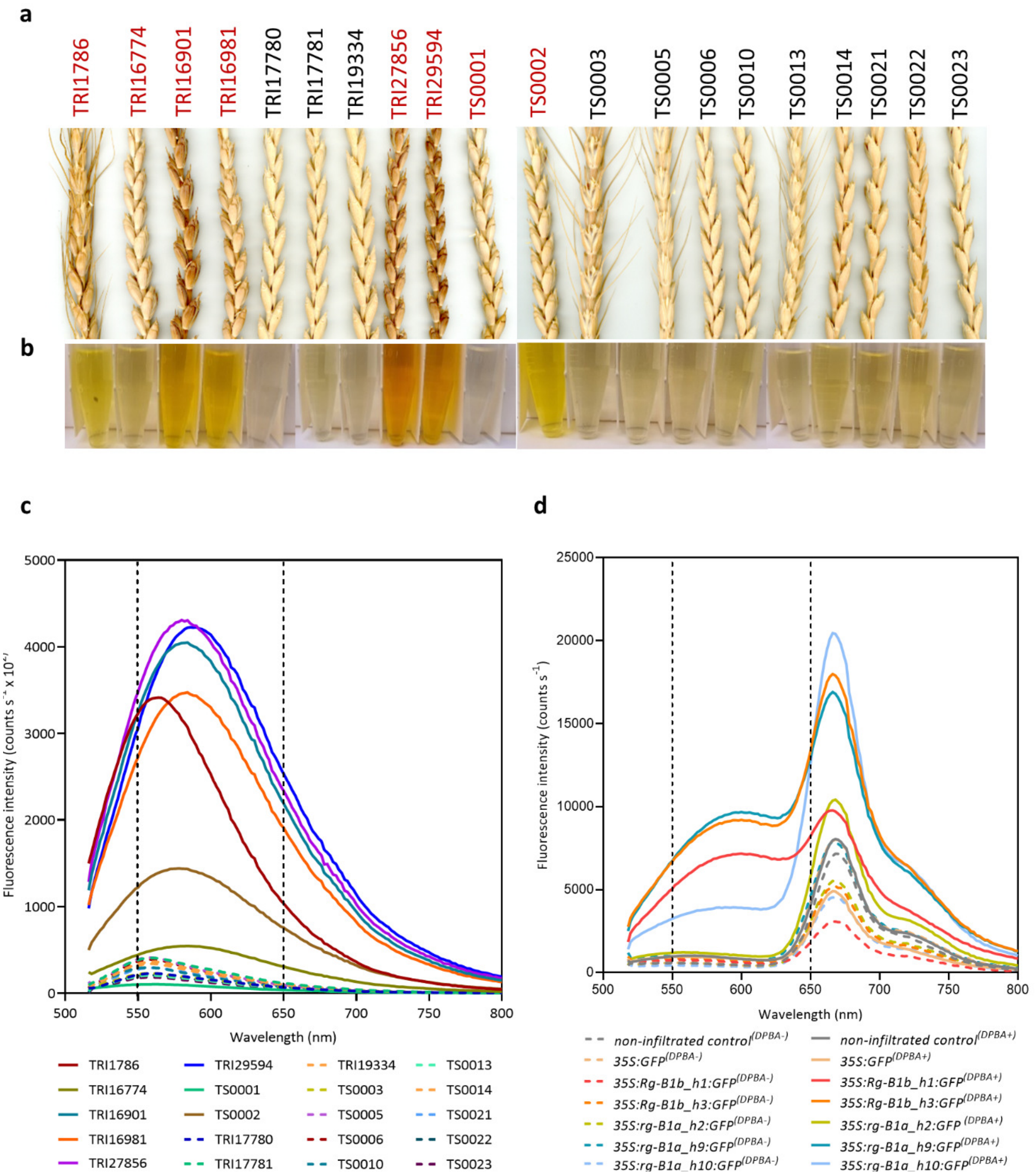

Supplementary Figure 12. Fluorescence intensity profile of spelt glumes. (**a**) Phenotype of 20 spelt accessions. (**b**) DPBA stained extract of spelt glumes. (**c**) Fluorescence intensity of DPBA stained extracts. Dotted lines indicate white accessions, solid lines red accessions. (**d**) Spectrofluorometric profile of agroinfiltrated leaves pre and post DPBA staining. The region between the two vertical dotted lines from 550-650 nm coincides with the previously reported peak for the flavonol quercetin. The peak from 665-685 nm is autofluorescence from chlorophyll/chloroplasts.

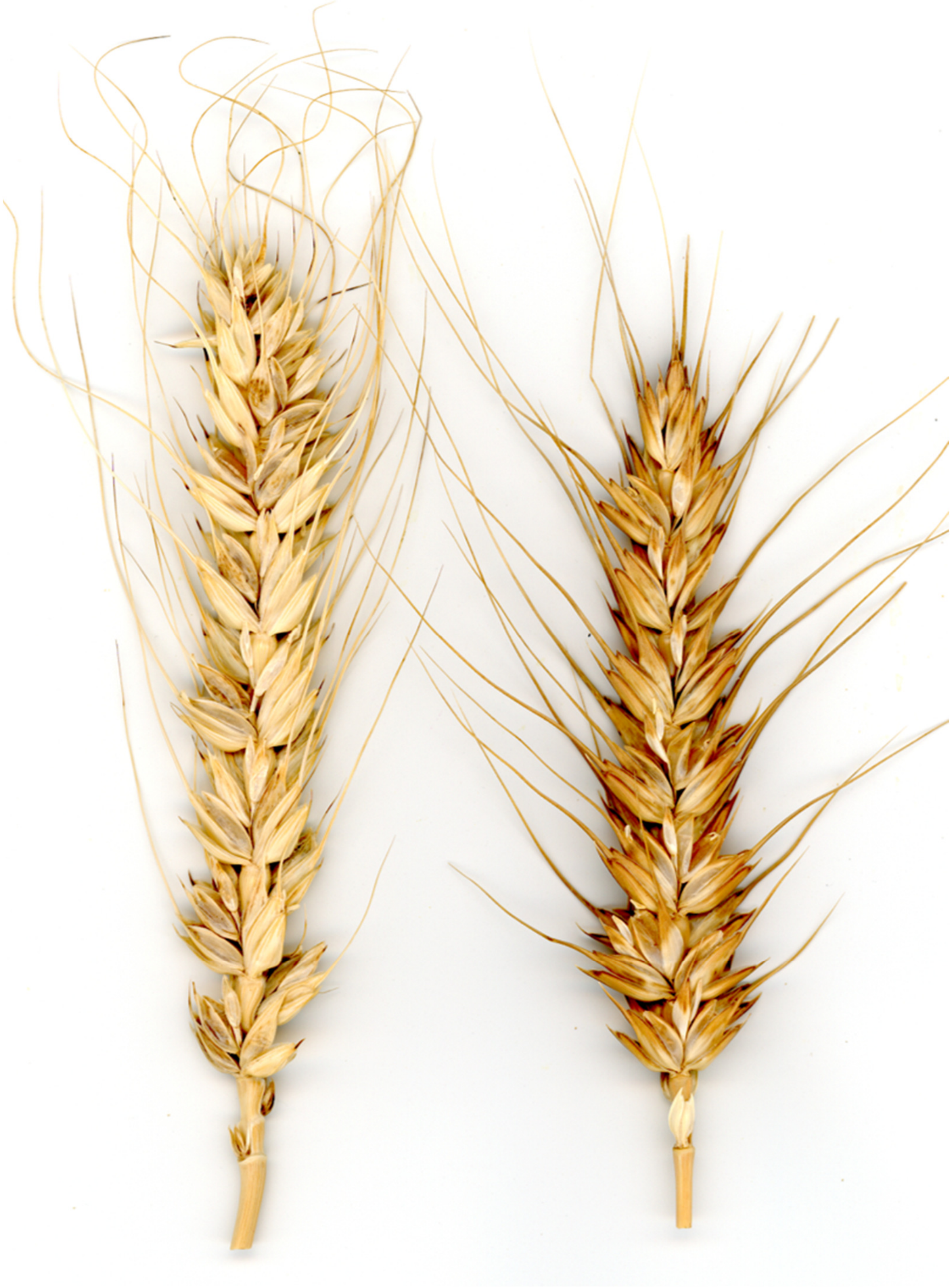

Supplementary Figure 13. Influence of light on the red glume phenotype. Shown are two spikes of the red glume wheat line TA5605. The spike on the left was covered with a pollination bag (left) while the spike on the right was not.

Supplementary Table 1. Parameter estimates for demographic modelling.

	**indepWestMig**		**westMig**		**westMigAdmix**	
	Ghost	Asian ancestral	Ghost	Asian ancestral	Ghost	Asian ancestral
N_{AS}	1,036,691	1,745,686	6,828,368	936,104	7,199,056	1,001,769
N_{CE}	5,274,951	908,584	2,976,393	2,295,759	2,702,867	1,503,286
N_{IB}	2,217,462	583,331	1,216,222	1,004,393	1,431,178	774,210
N_{ANC}	29,296,457	74,944,876	31,506,118	65,144,251	33,369,192	66,837,240
TBOTSTART	NA	8,121	NA	6,684	NA	8,444
TBOTEND	NA	8,000	NA	6,610	NA	8,357
NBOT	NA	672	NA	591	NA	598
$TFOUND_{AS}$	1,014	NA	5,588	NA	8,486	NA
$NFOUND_{AS}$	702	NA	627	NA	547	NA
$TFOUND_{IB}$	1,218	1,202	1,038	1,322	1,268	1,264
$NFOUND_{IB}$	605	320	395	318	443	265
$TFOUND_{CE}$	1,023	1,039	1,074	1,395	1,293	1,299
$NFOUND_{CE}$	967	458	662	409	696	362
R_{IB}	-0.0067	-0.0062	-0.0077	-0.0061	-0.0064	-0.0063
R_{CE}	-0.0084	-0.0073	-0.0078	-0.0062	-0.0064	-0.0064
R_{AS}	-0.0072	-0.0010	-0.0017	-0.0011	-0.0011	-0.0009
TMIG	NA	NA	NA	NA	958	1,103
NMIG	NA	NA	NA	NA	453	700

Parameter estimates and confidence intervals for the best performing model (indpWestMig)

Parameters	Point estimate	Q5%	Q95%
N_{AS}	1,036,691	871,467	4,762,619
N_{CE}	5,274,951	795,293	4,640,651
N_{IB}	2,217,462	1,676,913	3,720,851
N_{ANC}	29,296,457	19,541,580	23,011,428
$TFOUND_{AS}$	1,014	1,011	1,069
$NFOUND_{AS}$	702	439	777
$TFOUND_{IB}$	1,218	1,018	1,280
$NFOUND_{IB}$	605	438	617
$TFOUND_{CE}$	1,023	1,014	1,072
$NFOUND_{CE}$	967	328	816
R_{IB}	-0.0067	-0.0084	-0.0065
R_{CE}	-0.0084	-0.0085	-0.0074
R_{AS}	-0.0072	-0.0086	-0.0071

In the parameter names, subscripts indicate population names (AS: Asia, CE: Central Europe, IB: Iberia, ANC: ancestral); parameters beginning with N indicate population sizes, those beginning with T times, and those with R population growth rates (which are negative because in the coalescence framework inference goes backwards in time from the present to the past).

Supplementary Table 2. Demographic model comparisons.

**Model name**	**logL[a]**	**Nr. parameters[b]**	**AIC[c]**
indepWestMig_ghost	-7,758.08	10	**15,536.17**
westMig_ghost	-7,983.47	10	**15,986.94**
westMigAdmix_ghost	-8,081.42	12	**16,186.84**
indepWestMig_AsianAncestral	-9,045.23	11	**18,112.46**
westMig_AsianAncestral	-9,176.74	11	**18,375.49**
westMixAdmix_AsianAncestral	-9,349.66	13	**18,725.32**

[a] Log-likelihood of the model. [b]Number of parameters in the model. [c]Akaike information criterion, calculated as 2*Nr. parameters - 2*logL.

Supplementary Table 3. List of candidate gene in the *Rg1* interval based on the Chinese Spring RefSeq v.1.0

Gene id v1.1	Strand	Description
TraesCS1B02G003900	+	RuvB-like helicase 2
TraesCS1B02G004000	-	Disease resistance protein (TIR-NBS-LRR class) family
TraesCS1B02G004100	-	Receptor-like protein kinase
TraesCS1B02G004200	-	4-hydroxyphenylpyruvate dioxygenase
TraesCS1B02G004300	-	Tryptophan aminotransferase
TraesCS1B02G004400	-	Tryptophan aminotransferase
TraesCS1B02G004500	+	Glyoxylate reductase/hydroxypyruvate reductase
TraesCS1B02G004600	-	Disease resistance protein (NBS-LRR class) family
TraesCS1B02G004700	+	Receptor-kinase, putative
TraesCS1B02G004800	+	transmembrane protein, putative (DUF594)
TraesCS1B02G004900	+	Leucine-rich repeat receptor-like protein kinase family protein
TraesCS1B02G005000	-	Sentrin-specific protease
TraesCS1B02G005100	-	E3 ubiquitin-protein ligase SINA-like 10
TraesCS1B02G005200	+	Myb-like transcription factor
TraesCS1B02G005300	+	Hydroxycinnamoyl-CoA shikimate/quinate hydroxycinnamoyltransferase
TraesCS1B02G005400	-	Cyclotide
TraesCS1B02G005500	-	carboxyl-terminal peptidase, putative (DUF239)
TraesCS1B02G005600	+	SNF1-related protein kinase regulatory subunit beta-2
TraesCS1B02G006000	+	Flavin-containing monooxygenase
TraesCS1B02G006100	+	transmembrane protein, putative (DUF594)
TraesCS1B02G006200	+	Flavin-containing monooxygenase
TraesCS1B02G006300	+	Disease resistance protein (TIR-NBS-LRR class) family
TraesCS1B02G006400	+	F-box/LRR-repeat protein 14
TraesCS1B02G006500	+	Receptor protein kinase, putative
TraesCS1B02G006600	-	Charged multivesicular body protein 5
TraesCS1B02G006700	+	Receptor protein kinase, putative
TraesCS1B02G006800	+	Receptor protein kinase, putative
TraesCS1B02G006900	+	Disease resistance protein RPM1
TraesCS1B02G007000	+	Protein kinase family protein
TraesCS1B02G007100	+	Receptor protein kinase, putative
TraesCS1B02G007200	+	F-box/LRR-repeat protein
TraesCS1B02G007300	+	F-box/LRR-repeat protein
TraesCS1B02G007400	+	Leucine-rich repeat receptor-like protein kinase family protein, putative

Supplementary Table 4. List of *Rg-B1* alleles in ten high-quality wheat genomes.

Gene id	Wheat cultivar	*Rg-B1* allele	% coding sequence identity to *rg1-B1a_h1*	Group
TraesCS1B02G005200	Chinese Spring	*rg-B1a_h1*	100	Group 1
TraesSTA1B01G003600	CDC Stanley	*rg-B1a_h1*		
TraesTSP1B01G002900	PI 190962	*rg-B1a_h2*	99.2	
TraesJAG1B01G013700	Jagger	*rg-B1a_h2*		
TraesMAC1B01G003500	Mace	*rg-B1a_h2*		
TraesNOR1B01G004500	Norin 61	*rg-B1a_h2*		
TraesSTA1B01G002500	CDC Stanley	*rg-B1a_h2*		
TraesSYM1B01G002900	SY Mattis	*rg-B1a_h3*	99.1	
TraesLDM1B01G001500	CDC Landmark	*rg-B1a_h3*		
TraesLAC1B01G002500	Lancer	*rg-B1a_h3*		
TraesJUL1B01G000200	Julius	*rg-B1a_h3*		
TraesJAG1B01G012000	Jagger	*rg-B1a_h4*	99.3	
TraesNOR1B01G006200	Norin 61	*rg-B1a_h4*		
TraesTSP1B01G005500	PI 190962	*rg-B1a_h5*	99.6	
TraesMAC1B01G002800	Mace	*rg-B1a_h6*	96.2	
Unannotated	Jagger	*rg-B1a_h7*	97.0	Group 2
Unannotated	Norin 61	*rg-B1a_h7*		
Unannotated	PI 190962	*rg-B1a_h7*		
TraesJAG1B01G000800	Jagger	*Rg-B1b_h1*	97.1	Group 3
TraesNOR1B01G001100	Norin 61	*Rg-B1b_h1*		
TraesTSP1B01G005900	PI 190962	*Rg-B1b_h2*	97.0	
TraesTSP1B01G005700	PI 190962	*Rg-B1b_h3*	96.9	
TraesTSP1B01G005000	PI 190962	*rg-B1a_h8*	95.4	Group 4
TraesMAC1B01G005600	Mace	*rg-B1a_h9*	95.8	
TraesLAC1B01G000200	Lancer	*rg-B1a_h9*		
TraesTSP1B01G004800	PI 190962	*rg-B1a_h10*	93.1	Group 5

Supplementary Table 5. Amino acid identities of the different predicted Rg-B1 proteins.

	Rg-B1b_h1	Rg-B1b_h2	Rg-B1b_h3	rg-B1a_h1	rg-B1a_h2	rg-B1a_h3	rg-B1a_h4	rg-B1a_h5	rg-B1a_h6	rg-B1a_h7	rg-B1a_h8	rg-B1a_h9	rg-B1a_h10
Rg-B1b_h1	100												
Rg-B1b_h2	99.69	100											
Rg-B1b_h3	98.77	98.46	100										
rg-B1a_h1	95.06	94.75	94.44	100									
rg-B1a_h2	95.37	95.06	94.75	98.77	100								
rg-B1a_h3	95.37	95.06	94.75	98.77	100	100							
rg-B1a_h4	95.68	95.37	95.06	98.77	99.69	99.69	100						
rg-B1a_h5	95.99	95.68	95.37	99.07	99.38	99.38	99.69	100					
rg-B1a_h6	91.98	91.67	92.59	92.90	93.21	93.21	93.52	93.83	100				
rg-B1a_h7	91.80	91.80	92.62	92.21	92.62	92.62	93.03	93.44	91.80	100			
rg-B1a_h8	91.67	91.36	92.28	91.36	91.98	91.98	92.28	92.28	95.99	90.61	100		
rg-B1a_h9	92.90	92.59	93.52	92.59	92.90	92.90	93.21	93.52	94.44	90.20	95.37	100	
rg-B1a_h10	89.51	89.20	90.12	88.58	88.89	88.89	89.20	89.51	90.12	88.98	90.74	90.12	100
