# Supplementary material for: Population genomics and haplotype analysis in spelt and bread wheat identifies a gene regulating glume color
Source: Commun Biol. 2021 Mar 19;4:375. doi: 10.1038/s42003-021-01908-6 (PMC7979816; doi:10.1038/s42003-021-01908-6)
Supplement: Supplementary file 3 — Description of Additional Supplementary Files [file 42003_2021_1908_MOESM3_ESM.pdf]

Description of Additional Supplementary Files

**File Name:** Supplementary Data 1

**Description:** Passport information of the spelt and bread wheat accessions used in this study.

**File Name:** Supplementary Data 2

**Description:** Allele-specific Rg-B1 markers tested on 53 Central European spelt accessions.

**File Name:** Supplementary Data 3

**Description:** Glume color and Rg-B1 marker tested on 96 bread wheat accessions of the Japanese wheat collection (JWC).

**File Name:** Supplementary Data 4

**Description:** Marker – phenotype association in the TAM112 x Duster mapping population. Rg1 refers to the presence of the 334 bp amplicon specific for the functional Rg-B1 (group 3) alleles, while rg1 refers to absence of the 334 bp amplicon.

**File Name:** Supplementary Data 5

**Description:** List of primers used in this study.
