# Supplementary material for: Population genomics and haplotype analysis in spelt and bread wheat identifies a gene regulating glume color
Source: Commun Biol. 2021 Mar 19;4:375. doi: 10.1038/s42003-021-01908-6 (PMC7979816; doi:10.1038/s42003-021-01908-6)
Supplement: Supplementary file 9 — Reporting Summary [file 42003_2021_1908_MOESM9_ESM.pdf]

nature research

Corresponding author(s): Prof. Simon Krattinger

Last updated by author(s): Jan 26, 2021

Reporting Summary

Nature Research wishes to improve the reproducibility of the work that we publish. This form provides structure for consistency and transparency in reporting. For further information on Nature Research policies, see our Editorial Policies and the Editorial Policy Checklist.

Statistics

For all statistical analyses, confirm that the following items are present in the figure legend, table legend, main text, or Methods section.

n/a	Confirmed	
☐	☒	The exact sample size (*n*) for each experimental group/condition, given as a discrete number and unit of measurement
☐	☒	A statement on whether measurements were taken from distinct samples or whether the same sample was measured repeatedly
☐	☒	The statistical test(s) used AND whether they are one- or two-sided<br>*Only common tests should be described solely by name; describe more complex techniques in the Methods section.*
☒	☐	A description of all covariates tested
☒	☐	A description of any assumptions or corrections, such as tests of normality and adjustment for multiple comparisons
☒	☐	A full description of the statistical parameters including central tendency (e.g. means) or other basic estimates (e.g. regression coefficient) AND variation (e.g. standard deviation) or associated estimates of uncertainty (e.g. confidence intervals)
☐	☒	For null hypothesis testing, the test statistic (e.g. *F*, *t*, *r*) with confidence intervals, effect sizes, degrees of freedom and *P* value noted<br>*Give P values as exact values whenever suitable.*
☒	☐	For Bayesian analysis, information on the choice of priors and Markov chain Monte Carlo settings
☒	☐	For hierarchical and complex designs, identification of the appropriate level for tests and full reporting of outcomes
☒	☐	Estimates of effect sizes (e.g. Cohen's *d*, Pearson's *r*), indicating how they were calculated

Our web collection on statistics for biologists contains articles on many of the points above.

Software and code

Policy information about availability of computer code

Data collection	Triticum aestivum genomes and annotations were downloaded from [https://wheat-urgi.versailles.inra.fr/] and [https://wheat.ipk-gatersleben.de/].
Data analysis	Population analysis were performed using bwa v0.7.15, TASSEL v5.2.31, vcftools v0.1.14, scikit-learn v0.17.1, SNPhylo software, SNPRelate package, MUSCLE, PHYLIP package, phangorn package, FigTree v1.4.3, ADMIXTURE software and PLINK v1.9<br>Demographic inference analysis were performed with fastSimcoal2 v2.6.0.3, DaDi<br>GWAS was performed using PLINK v1.9 and qqman R package<br>The detection of MYB copies was performed using BLAST v2.6.0, exonerate v2.2.0 and MEGA-X software

For manuscripts utilizing custom algorithms or software that are central to the research but not yet described in published literature, software must be made available to editors and reviewers. We strongly encourage code deposition in a community repository (e.g. GitHub). See the Nature Research guidelines for submitting code & software for further information.

Data

Policy information about availability of data

All manuscripts must include a data availability statement. This statement should provide the following information, where applicable:

- Accession codes, unique identifiers, or web links for publicly available datasets
- A list of figures that have associated raw data
- A description of any restrictions on data availability

Plant material were obtained from Agroscope (BDN, Switzerland) [https://www.bdn.ch/organisations/acw/], IPK (IPK Gatersleben, Germany) [http://www.ipk-

gatersleben.de/en/genebank/], CGN (WUR, Wageningen, Netherlands) [https://www.wur.nl/en/Research-Results/Statutory-research-tasks/Centre-for-Genetic-Resources-the-Netherlands-1.htm], ARS-GRIN (USDA, USA) [https://www.ars-grin.gov/]

The raw sequence reads were deposited in the short read archive (SRA) on NCBI under the accession PRJNA498918.
The VCF file is available on DRYAD repository under [https://doi.org/10.5061/dryad.d7wm37pzs]

Field-specific reporting

Please select the one below that is the best fit for your research. If you are not sure, read the appropriate sections before making your selection.

- [x] Life sciences
- [ ] Behavioural & social sciences
- [ ] Ecological, evolutionary & environmental sciences

For a reference copy of the document with all sections, see nature.com/documents/nr-reporting-summary-flat.pdf

Life sciences study design

All studies must disclose on these points even when the disclosure is negative.

Sample size	75 T. aestivum ssp. aestivum and 267 T. aestivum ssp. spelta accessions from Central Europe, Southern Europe, Africa, Asia, and America were studied for population analysis.<br>102 Central European spelt accessions were used for GWAS analysis for red glume color (Rg1) trait<br>53 Central European spelt accessions were used for validating the Rg1 markers<br>20 Central European spelt accessions were used for flavonoid structural gene expression studies
Data exclusions	Wheat-x-Spelt, African, and American accessions were discarded from the demographic inference analysis as the studies focus on Asia and Europe.<br>All the spelt accession which don't have red glume phenotype information were discarded for the GWAS analysis.
Replication	Three biological and three technical replicates were used for all the RT-qPCR experiments.<br>Transient expression in Nicotiana benthamiana was performed in three replicates for each construct in every experiment. Fluorescence measurement of DPBA stained and unstained extract was performed in three technical replicates.<br>Agroinfiltration, DPBA staining and fluorescence measurement experiments were repeated at-least two times to ensure reproducibility.
Randomization	The Central European spelt accessions representing both red and white glume colour were randomly selected marker validations and gene expression studies.
Blinding	*Describe whether the investigators were blinded to group allocation during data collection and/or analysis. If blinding was not possible, describe why OR explain why blinding was not relevant to your study.*

Reporting for specific materials, systems and methods

We require information from authors about some types of materials, experimental systems and methods used in many studies. Here, indicate whether each material, system or method listed is relevant to your study. If you are not sure if a list item applies to your research, read the appropriate section before selecting a response.

Materials & experimental systems

n/a	Involved in the study
[x]	[ ] Antibodies
[x]	[ ] Eukaryotic cell lines
[x]	[ ] Palaeontology and archaeology
[x]	[ ] Animals and other organisms
[x]	[ ] Human research participants
[x]	[ ] Clinical data
[x]	[ ] Dual use research of concern

Methods

n/a	Involved in the study
[x]	[ ] ChIP-seq
[x]	[ ] Flow cytometry
[x]	[ ] MRI-based neuroimaging

April 2020
